# ERRATUM

**DOI:** 10.2174/1570159X1304150831121355

**Published:** 2015-07

Due to an overlook on the author's side, author’s name in the article entitled as: “**Caffeine: Cognitive and Physical Performance Enhancer or Psychoactive Drug?**” by Dr. Daria Piacentino (Co-author) published in the journal “*Current Neuropharmacology*” Volume 13, No 1, Page no 71-88, was wrong. The correct name is as follows:

Simone Cappelletti^1^, Daria Piacentino^2^, Gabriele Sani^2^ and Mariarosaria Aromatario^1,*^. Caffeine: Cognitive and Physical Performance Enhancer or Psychoactive Drug. *Curr. Neuropharmacol.,* **2015**, *13*(1), 71-88.

